# VACCINES OVER ANTIBIOTICS: SAUDI ARABIA’S JOURNEY OF MANAGING PEDIATRIC BACTERIAL MENINGITIS

**DOI:** 10.21010/Ajidv19i2S.6

**Published:** 2025-10-17

**Authors:** ALNASSER Yossef, BIN OBAID M. Nada, BAMAREI M. Noura, ALSHEHRI S. Aseel, JAKHETE A. Akhilesh, BINKHAMIS Khalifa

**Affiliations:** * Pediatric Department, Bronxcare Health System, New York, NY, United States; † College of Medicine, King Saud University, Riyadh, Saudi Arabia; †† Geroge Washington Milken School of Public Health, Washington, DC, United States; ** Microbiology Unit, Department of Pathology, College of Medicine, King Saud University, Riyadh, Saudi Arabia

**Keywords:** Saudi Arabia, Bacterial Meningitis, Vaccines, Antibiotics, Drug Resistance

## Abstract

**Background:**

Saudi Arabia had high rates of bacterial meningitis in the late 90s. Children are at highest risk of this devastating disease with poor outcomes.

**Objective:**

The study aims to evaluate the prevalence, causative pathogens, and antibiotic resistance patterns in pediatric bacterial meningitis cases at a tertiary hospital in Riyadh, Saudi Arabia.

**Materials and Methods:**

Single-center retrospective chart review and cross-sectional methodology was conducted at King Saud University Medical City (KSUMC) from 2015 to 2023.

**Result:**

Reviewing 8 years of CSF culture results only yielded 37 cases. This is only 0.5% of total hospital admissions over 8 years. The majority of cases were for children under the age of 2 years (82%). Gender of cases was almost equal and there was no seasonal variation. The most common organisms were gram-positive (14, 38%) including Group B streptococcus (GBS) (4, 11%), *Streptococcus pneumoniae* (4, 11%). Gram-negative organisms caused 8 cases (22%) by 5 different organisms. There was no Haemophilus influenza type B or meningococcus found in any of the CSF cultures. The single sample of *Staphylococcus aureus* was methicillin-resistant *Staphylococcus aureus* (MRSA) and three gram-negative organisms were multidrug resistant.

**Conclusion:**

Saudi Arabia provides an example of the success of a mass vaccination program to curb the burden of pediatric bacterial meningitis. Future efforts should focus on antibiotic stewardship, mass screening of GBS, and adopting additional strains for the pneumococcus vaccine. Further research is needed to address the rising number of gram-negative organisms causing pediatric bacterial meningitis in Saudi Arabia and globally.

## Introduction

Pediatric Meningitis can be a life-threatening inflammatory condition affecting the protective layers of a growing brain and spinal cord. It could be caused by bacterial, viral, or fungal pathogens (Al-Binali and Al-Fifi., 2002). However, bacterial infections are very serious, with possible long-lasting sequences (Van De Beek *et al.*, 2016). With the high burden and cost of managing pediatric bacterial meningitis, prevention is key to addressing this serious public health issue.

Saudi Arabia had a high burden of pediatric meningitis in the past (Al-Binali and Al-Fifi., 2002) Some of the risks of pediatric meningitis can be attributed to the large number of Muslim pilgrims participating in Hajj and Umrah in small spaces, allowing human-to-human transmission (Yezli *et al.*, 2016). Few studies were found about the epidemiology of pediatric meningitis in Saudi Arabia, most of which were conducted 20-30 years ago. Almazrou *et al.*, (2004) reported 208 cases across five cities over two years involving children under the age of five years (Almazrou *et al.*, 2004) During the same period, Almuneef *et al.*, (1998) documented 70 children diagnosed with bacterial meningitis (Almuneef *et al.*, 1998).

Studies in the 90s and early 2000s before mass vaccination program was implemented had identified *Haemophilus influenzae type b* (Hib) as the most common organism causing meningitis in the region (Al-Binali and Al-Fifi., 2002, Yezli *et al.*, 2016) Similarly, Hib cases were predominant in Saudi Arabia before the introduction of the Hib vaccines into the regular immunisation schedule (Almuneef *et al.*, 1998, Azubuike, 1990. Then, Saudi policymakers felt obligated to act to protect Muslim pilgrims and the nation’s children.

Even though early treatment with antibiotics had been shown to improve pediatric bacterial meningitis outcomes, prevention should be the primary focus due to the serious consequences of this disease (Alamarat and Hasbun, 2020). Additionally, the emergence of drug resistance among bacteria adds a new challenge. Albinali and Alfifi found high rates of resistant organisms among a cohort of pediatric meningitis in the southern province of Saudi Arabia (Al-Binali and Al-Fifi., 2002). One of the major contributors to the emerging antibiotic resistance in Saudi Arabia was uncontrolled antibiotic prescription, with their availability over-the-counter in the past (Zowawi, 2016). Now, the Saudi Ministry of Health regulates antibiotic prescription and penalised pharmacies for dispensing antibiotics without prescription in recent years.

With emerging antibiotic resistance, depending on secondary prevention cannot solve this public health threat to the children and youths. Adopting primary prevention can prevent pediatric bacterial meningitis and preserve children’s neurodevelopment for a better future. One of the essential and cost-effective primary prevention measures for pediatric bacterial meningitis is mass vaccination (Zainel *et al.*, 2021). The Saudi vaccine program was established 30 years ago as an essential part of primary health care (Tufenkeji and Kattan, 1994). The vaccination program has effectively decreased mortality and morbidity in childhood infectious diseases (Alabadi and Aldawood, 2020). In particular, the Saudi government mandated a mass vaccination program to protect all children and Muslim pilgrims in the hope of preventing pediatric meningitis in the young Kingdom (Yezli *et al.*, 2016).

This novel study aims to identify the most common organisms causing bacterial meningitis after decades of successful implementation of mass vaccination programs. This can help in assessing the effectiveness of the already implemented preventive measures. Moreover, this analysis can guide policymakers in determining the need for additional vaccines and expanding the current program. Furthermore, the study aims to evaluate the burden of multidrug resistance organisms (MDRO) on pediatric bacterial meningitis in recent years after a decade of antibiotic regulation in Saudi Arabia. MDRO in Saudi Arabia have a high potential of spreading globally due to mass gathering and the melting pot nature of the country among the Islamic world.

## Materials and Methods

### Study Design and Participants

This study adopted the retrospective chart review approach and cross-sectional methodology. It involved chart reviews of electronic medical records of pediatric patients at King Saud University Medical City (KSUMC). Single-center and cross-sectional methodologies were chosen as the first step to identify the problem and set the stage for more robust research in the future. Inclusion criteria are children aged 0-16 years treated at KSUMC from May 2015 to August 2023 who had positive cerebrospinal fluid (CSF) bacterial culture; patients with positive CSF or partially treated meningitis. Partially treated meningitis was defined as any antibiotic exposures prior to obtaining CSF sample. All samples, first-time or recurrent, were included. Pediatric patients who had a negative CSF culture or were ruled out clinically of having meningitis were not included.

### Data Collection

Data were collected from pediatric patients’ electronic records who met the inclusion criteria. Clinical and demographic data were collected with no patient identifiers. Later, microbiological and antibiotic susceptibility patterns were matched with clinical and demographic data. Missing data were supplemented through extensive chart review of initial hospital admissions and follow-up clinical notes. To ensure the quality of data collection, each datum entry was reviewed by two investigators. Contaminated samples were confirmed by an experienced microbiologist and the principal investigator.

## Statistical Analysis

Data were coded without any patient’s identifier and listed in an Excel sheet. Then, Data analysis was conducted utilizing SAS (version 9.4, Carey, NC) Mean, median, and standard deviation were generated for continuous variables, and frequency distributions were generated for all categorical data. The model selection technique was used to assess if any of the clinical factors could be used as predictors to diagnose a case of pediatric bacterial meningitis. In this case, a backward selection technique with a cutoff of 0.05 to identify the most important factors was utilized. The backward model selection is a statistical modeling technique in which the initial model includes all the variables. Step by step, the variables get eliminated if they were found not significant at a predetermined level (alpha) (Király and Hangya, 2022). In this study, we selected an alpha value of 0.05 which is the most commonly used value in statistics. A logistic regression model was generated to identify any association between clinical parameters and demographic data such as age, vaccination status, development status, and seasonal variations, among other factors. Confounders and effect modifiers were tested as well.

Effect modification was handled in the analysis phase by including an interaction term in the model. After running the model, if the interaction term was statistically significant at 0.05 level, we would conclude that effect modification is present.

### Study Ethics

Study approval was obtained from the research ethics committee at King Saud University with number E-23-8288 prior to any data collection. Data were coded, and patients’ identifiers were removed. Data were stored and encrypted by the principal investigator. The study followed international ethical standards (e.g., the Declaration of Helsinki) and local institutional regulations.

## Results

### Participants’ Characteristics and Demographics

After reviewing eight years of positive CSF culture results, only 37 CSF samples had bacteria growth. Out of the 37 CSF samples, 22 had a true bacterial infection, while the remaining 15 were thought to be contaminants. The majority of cases were under 2 years of age (82%, 30), with over 50% of all cases being infants. Most patients were born full-term, while 19% were preterm. The study group had a slightly higher male representation as compared to female. **Table 1**.

**Table 1:** Demographics of enrolled patients

n= 37		Frequency (%)
Age	<1 year	23(62.16)
	1-2 years	7(18.92)
	3-5 years	1(2.70)
	6-12 years	6(16.22)
	13-16 years	0(0)

Gender	Male	21(56.76)
	Female	16(43.24)

Birth status	Full-term	26(70.27)
	Preterm	7(18.91)
	Post term	2(5.41)
	Don’t know	2(5.41)

### Bacterial Etiologies

**Figure 1:**
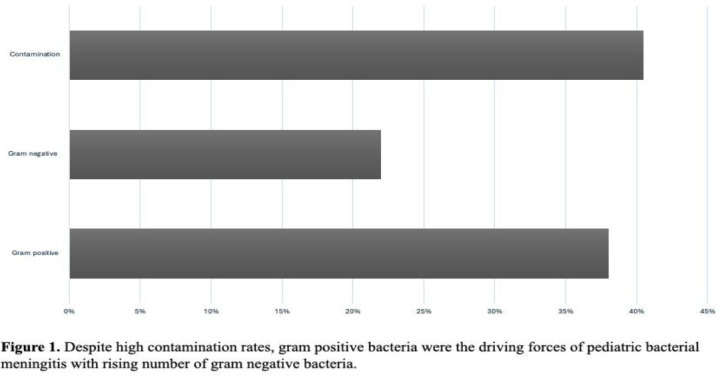


The most common bacterial etiology was gram-positive (14, 38%), including Group B streptococcus (GBS) (4, 11%), *Streptococcus pneumoniae* (4, 11%), *Enterococcus faecalis* (2, 4%), *Staphylococcus aureus* (1, 3%), *Microbacterium species* (1, 3%), *Staphylococcus hominis* (1, 3%) and *Lactococcus species* (1, 3%). **Figure 1** In contrast, gram-negative constituted eight samples (22%) including *Pseudomonas aeruginosa* (3, 7%), *Pseudomonas putida* (1, 3%) *Citrobacter sedlakii* (1, 3%), *Enterobacter cloacae* (1, 3%), *Salmonella* (1, 3%), *Escherichia coli (E. coli)* (1, 3%). There was a high contamination rate among the reported CSF cultures (15, 40.5%). None of the CSF samples grew Hib or meningococcus during the study period.

### Seasonal Variation, Vaccination Status, and Burden of Disease

**Figure 2:**
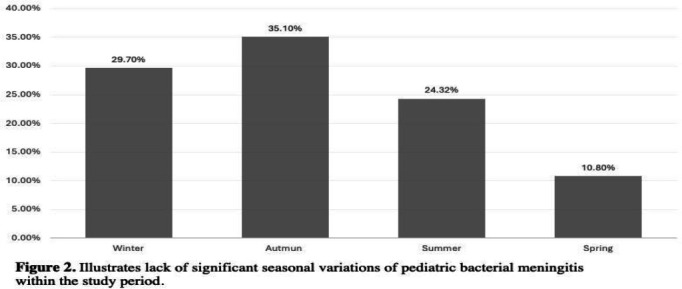


The study has found no seasonal variation, and meningitis occurred in all four seasons, with non-statistically significant higher incidence in winter and autumn. **Figure 2** Majority of included children have up-to-date vaccination status according to their age (75%), with only 10% not meeting their vaccination schedule for their age. Thus, pediatric meningitis admissions represented 0.5% of the total hospital admissions to pediatrics in KSUMC over the past eight years.

### Antibiotic Resistance Patterns

GBS was pan-susceptible with no reported resistance. *Streptococcus pneumoniae* showed similar patterns. *Staphylococcus aureus* showed 100% susceptibility to vancomycin and linezolid but was methicillin-resistant (MRSA). *Microbacterium species* showed no significant resistance. Similarly, *Enterococcus faecalis* and *Lactococcus species* followed the same pathway. *Pseudomonas aeruginosa* showed resistance of 66.66% to ceftazidime, cefepime, and piperacillin/ tazobactam but was susceptible to meropenem and tobramycin. On the other hand, *Enterobacter cloacae* showed good susceptibility to antibiotics with gram-negative coverage. Meanwhile, *Salmonella* was 100% resistant to ampicillin, gentamycin, amikacin, and trimethoprim/sulfamethoxazole but susceptible to ceftriaxone/cefotaxime, ceftazidime, cefepime, and meropenem. Lastly, *E. coli* was 100% resistant to ampicillin, ceftriaxone/cefotaxime, ceftazidime, and cefepime while 100% susceptible to meropenem, gentamycin, amikacin, trimethoprim/sulfamethoxazole **Table 2**.

**Table 2:** Antibiotic resistance and sensitive pattern of organisms isolated from CSF samples of children at a tertiary hospital in Riyadh, Saudi Arabia

Organisms	Antibiotics	Resistance	Sensitive
Group B streptococcus (agalactiae) (n=4)	Penicillin	-	100%
	Ampicllin	-	100%
	Ceftriaxone/Cefotaxime	-	100%
	Vancomycin	-	100%
*Streptococcus Pneumonia* (n=4)	Penicillin	-	100%
	Ceftriaxone/Cefotaxime	-	100%
	Vancomycin	-	100%
*Staphylococcus aureus* (n=1)	Oxacillin	100%	-
	Vancomycin	-	100%
	Linezolid	-	100%
Microbacterium species (n=1)	Penicillin	-	100%
	Ceftriaxone	-	100%
	Meropenem	-	100%
	Vancomycin	-	100%
*Enterococcus faecalis* (n=2)	Ampicllin	-	100%
	Vancomycin	-	100%
	Linezolid	-	100%
	Gentamicin synergy	-	100%
	Streptomycin synergy	-	100%
*Lactococcus species* (n=1)	Penicillin	-	100%
	Ceftriaxone	-	100%
	Vancomycin	-	100%
*Staphylococcus hominis* (n=1)	Oxacillin	100%	-
	Vancomycin	-	100%
	Linezolid	-	100%
*Pseudomonas aeruginosa* (n=3)	Ceftazidim	33.33%	66.66%
	Cefipime	33.33%	66.66%
	Meropenm	-	100%
	Tobramycin	-	100%
	Piperacillin/ Tazobactam	33.33%	66.66%
*Pseudomonas putida* (n=1)	Ceftazidim	-	100%
	cefipime	-	100%
	Meropenm	-	100%
	Tobramycin	-	100%
*Citrobacter sedlakii* (n=1)	Ampicillin	100%	
	Ceftriaxone/Cefotaxime		100%
	Ceftazidime		100%
	Cefipime		100%
	Meropenem		100%
	Gentamicin synergy		100%
	Amikacin		?
	Trimethoprim/Sulfamethoxazole		?
*Entrobacter cloacea* (n=1)	Ampicllin	100%	-
	Ceftriaxone/Cefotaxime	-	100%
	Ceftazidim	-	100%
	Cefipime	-	100%
	Meropenm	-	100%
	Gentamicin synergy	-	100%
	Amikacin	-	100%
	Trimethoprim/Sulfamethoxazole	-	100%
*Salmonella species* (n=1)	Ampicllin	100%	-
	Ceftriaxone/Cefotaxime	-	100%
	Ceftazidim	-	100%
	Cefipime	-	100%
	Meropenm	-	100%
	Gentamicin synergy	100%	-
	Amikacin	100%	-
	Trimethoprim/Sulfamethoxazole	100%	-
*E.coli* (n=1)	Ampicllin	100%	-
	Ceftriaxone/Cefotaxime	100%	-
	Ceftazidim	100%	-
	Cefipime	100%	-
	Meropenm	-	100%
	Gentamicin synergy	-	100%
	Amikacin	-	100%
	Trimethoprim/Sulfamethoxazole	-	100%

### Factors Associated with Higher Risk of Pediatric Bacterial Meningitis

A variety of clinical parameters were measured to find any correlation between the clinical features and the likelihood of developing pediatric meningitis among Saudi children. There was an increase in the risk of having meningitis among the unvaccinated patients by 66%. This increment was correlated to odds ratio of 1.66 (95% CI 0.28–9.68). The risk was higher by 43% in the partially vaccinated children based on an odds ratio of 1.43 (95% CI 0.07–28.8). There were higher odds of being truly infected with a gram-negative pathogen. After eliminating all contamination, the odds of being infected with gram-negative organisms were 12 times higher (OR 20.13, CI 95% 1.11-365.94) than gram-positive pathogens (OR 7.97, CI 95% 0.65-97.2). There was no correlation discovered between pediatric meningitis and certain demographic characteristics, including age, gender, immune status, prematurity, seasonality, or the presence of comorbidities.

## Discussion

 Among children, meningitis is a life-threatening infectious disease that can lead to a variety of complications. A few studies conducted in Saudi Arabia in the 90s and early 2000s documented a high prevalence of pediatric bacterial meningitis caused mainly by *Hib, pneumococcal*, and *Neisseria meningococcus* (Al-Binali and Al-Fifi 2002, Al-Mazrou *et.al* 2004, Almuneef *et.al* 1998). Furthermore, meningitis outbreaks erupted in Saudi Arabia in that era and were linked to cases that attended the Hajj and Umrah pilgrimage in 1987, 1992, 2000, and 2001 (Badur *et al.*, 2021). Besides Hajj and Umerah, the proximity of Saudi Arabia to Africa, an endemic area for meningitis, places the country at constant risk. With the high risk and burden of the disease, Saudi policymakers had to act. They were an early adopter of mandated mass vaccination programs addressing the three main organisms. This mass vaccination was implemented in 2002 for citizens and residents while adding it as an entry requirement for hajj and Umrah to cover meningococcus, Hib and pneumococcus (Badur *et al.*, 2021, Al-Zamil, 2008, Khalil *et al.*, 2000) . The Saudi public accepted this mass vaccination due to their high trust in the Saudi Ministry of Health and mandating it for schools (Alnasser *et al.*, 2021).

Today, the Saudi government, policymakers, and the public are harvesting this successful implementation of mass vaccination to control this devastating disease. The low number of cases over eight years in our findings is a true testimony of such public health accomplishment. From the start, the Saudi routine childhood vaccination program was deemed to be successful. Even before expanding the program to its current status, it was able to lower the burden of pediatric bacterial meningitis after the introduction of one vaccine. Soon after starting the Hib vaccine, a study by Al-Mazrou *et al*. (2004) documented a decline in the incidence of pediatric bacterial meningitis from 10 to 2 per year (Almuneef *et al.*, 1998).This is a huge reduction of 80%. Worldwide, meningitis due to serogroup A of *Neisseria meningococcus* (NmA) has substantially decreased since 2010, with the progressive introduction of a meningococcal serogroup A conjugate vaccine. NmA is the most common type of meningococcus in Africa, meningitis belt to be specific (Reese *et al.*, 2019). Saudi Arabia mirrored other nations in lowering the burden of pediatric bacterial meningitis after the introduction of the NmA conjugate vaccine (Memish *et al.*, 2013). This success highlights the importance of vaccination in controlling pediatric bacterial meningitis, even in Saudi Arabia, a country with a high incidence rate of primary immunodeficiency (Al-Mousa and Al-Saud, 2017).

*GBS* is a type of bacteria that can be transmitted from mother to newborn during childbirth, potentially leading to serious conditions such as sepsis or meningitis (Winn and Winn 2021). Our study showed that *GBS* was one of the most common organisms causing pediatric bacterial meningitis. A systemic review conducted to estimate the prevalence of *GBS* colonization in Saudi Arabia found that the prevalence among pregnant women varied widely and ranged from 2.1% to 32.8% (Alshengeti, 2022). A retrospective chart review of over 13 years in a tertiary hospital in Riyadh found that the incidence of newborn *GBS* infection in Saudi Arabia is equivalent to the worldwide incidence rate (Al Luhidan *et al.*, 2019). This contradicts the common misconception and current practice about the role of *GBS* on Saudi infants’ morbidity. Our findings join earlier advocates in the value of screening and treating *GBS* to prevent and detect neonatal sepsis and pediatric bacterial meningitis (Alshengeti, 2022, Al Luhidan *et al.*, 2019, Surrati *et al.*, 2024).

It is alarming that gram-negative bacteria are playing higher roles in causality of pediatric bacterial meningitis. It is well-known that gram-negative bacterial meningitis has a worse prognosis and more complications (Pomar *et al.*, 2013). The rise of gram-negative bacteria is noted not only in Saudi Arabia. Many developed and developing countries report similar patterns (Hallmaier-Wacker *et al.*, 2022). This has a significant impact on global child health and a true public health concern around the world. New prevention tools need to be implemented to halt the rise of gram-negative bacteria causing pediatric bacterial meningitis.

Gram-negative bacteria not only cause devastating outcomes of pediatric bacterial meningitis, but they are also becoming resistant to antibiotics, especially *E. Coli* (Erb *et al.*, 2007). This was reflected in our findings as well. The rising multi-drug resistance (MDR) seen in this study can be linked to the Saudi population’s historical ability to get antibiotics without a prescription. Self-medication with over-the-counter antibiotics and the abuse of antibiotics, even by physicians, are possibly the causes of this problem (Zowawi, 2016, Al Harbi *et al.*, 2023). This issue is not limited to Saudi Arabia; it is a public health concern in the Middle East and many developing countries at large. Another study from Egypt found 36.6% of bacterial isolates were multi-drug resistant, with a significant portion being gram-negative organisms (Abdelkader *et al.*, 2017). The MDR threatens the health of the whole world and is not limited to *E. Coli*. Previous research has demonstrated resistance to *Streptococcus pneumoniae* and Hib (Al-Binali and Al-Fifi 2002, Almuneef *et al.*, 1998). Even the lone *Staphylococcus aureus* was found to be community-acquired MRSA. Community-acquired MRSA is a huge public health threat in Saudi Arabia and the whole region (Bukharie, 2010). Luckily, Saudi policymakers have established new rules and regulations to control the overuse of antibiotics and fight MDR bacteria. More work needs to be done to halt the rise of MDR around the globe.

Though CSF is highly accessible to diagnose meningitis, contamination of the CSF culture is a major concern (Garg, 2020). In this study, 40% of the isolates were deemed to be contaminated.

When obtaining CSF samples, strict hygiene standards must be followed to prevent contamination that can mask the growth of actual pathogens in the culture. By establishing better infection control and precautions while collecting CSF culture, contamination rates can be decreased, and the culture findings can be more accurately obtained. This makes our findings a call for action to improve the sterile environment while performing lumbar puncture.

Our study suffers from multiple limitations. The small sample size and single-center nature can limit the generalizability and may not yield sufficient data to reach reliable conclusions. Although this study was conducted in a tertiary hospital in the capital city, it remains a single-center study. Another limitation was in the study design as a retrospective chart review. The data’s quality and details were dependent on the quality of the documentation. Certain variables like consanguinity, maternal age, maternal level of education, and family income were not collected due to a lack of documented data in many charts. Another major limitation is in the high contamination rate which can impact the internal validity of the study. A better prospective design with more emphasis on infection control and sterile technique should be addressed in future research.

## Conclusion

After over two decades of mandated routine mass vaccination for all children in Saudi Arabia, the burden and incidence of pediatric bacterial meningitis halted. This can provide an example to many neighboring and developing countries to address the burden of pediatric bacterial meningitis. Furthermore, the etiologies of pediatric bacterial meningitis in Saudi Arabia differ compared to the 90s profile. In recent years, Saudi Arabia was able to reduce HiB and meningococcus from causing pediatric bacterial meningitis. It is time to start addressing GBS and implement mass screening for all pregnant women in Saudi Arabia. Expanding pneumococcal vaccines to add more strains is advisable. The rise of gram-negative bacteria in causing pediatric bacterial meningitis warrants further research to address this new threat. At the same time, continuing efforts to tackle emerging MDRO should be augmented locally and globally.

**Conflict of Interest:** The Authors declare that there is no conflict of interest associated with this study.

**List of Abbreviations:**
Hib: Haemophilus influenzae type b;
MDRO: Multidrug resistance organisms;
CSF: Cerebrospinal fluid;
GBS: Group B streptococcus;
MRSA: Methicillin-resistant *Staphylococcus aureus*
NmA: Serogroup A of *Neisseria Meningococcus*
